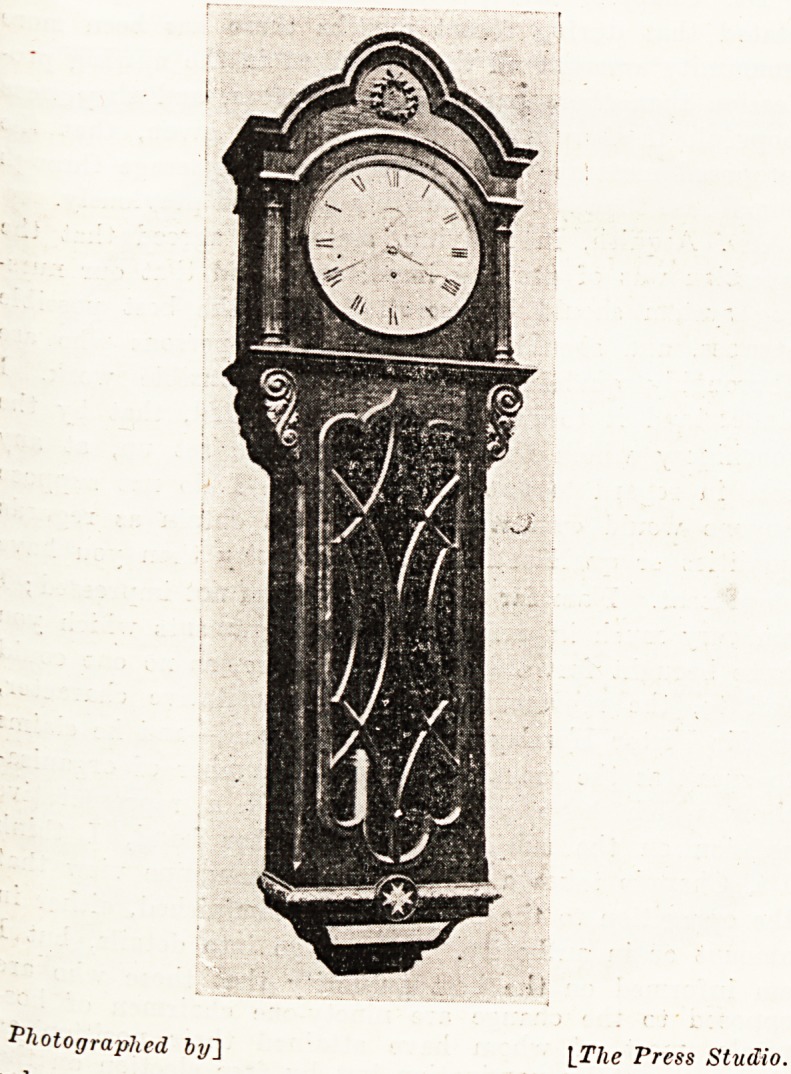# The Twining Memorial Clock

**Published:** 1913-05-03

**Authors:** 


### The Twining Memorial Clock.

A fund privately raised, and amounting to £248, has been subscribed in memory of the late Miss Louisa Twining, of which £200 has been given for a memorial bed named after her at Chalfont Colony for Epileptics. Part of the rest has been spent on a clock, of which an illustration is appended, which now hangs in the staff dining-hall of the same institution. Of dark polished oak and over 12 feet long, it is guaranteed by Bensons, Ludgate Hill, the

*Photographed by*] [*The Press Studio.*

makers, to go for one hundred years. Above the face is a wreath of laurel in relief, and below the pendulum, by permission, the Order of Lady of Grace of St. John of Jerusalem enlarged, with the lions in silver and the Cross in white. The inscription is "Louisa Twining, 1820-1912, Lady of Grace of the Order of St. John of Jerusalem in England." The remainder of the fund will go to the Women's Local Government Society, of which Miss Twining was vice-president.

## Figures and Tables

**Figure f1:**